# Spontaneous creation and annihilation dynamics and strain-limited stability of magnetic skyrmions

**DOI:** 10.1038/s41467-020-17338-7

**Published:** 2020-07-15

**Authors:** Frederic Rendell-Bhatti, Raymond J. Lamb, Johannes W. van der Jagt, Gary W. Paterson, Henk J. M. Swagten, Damien McGrouther

**Affiliations:** 1 SUPA, School of Physics and Astronomy, University of Glasgow, Glasgow, G12 8QQ UK; 2 Department of Applied Physics, Eindhoven University of Technology, 5612 AZ Eindhoven, The Netherlands

**Keywords:** Topological defects, Information storage, Magnetic properties and materials, Imaging techniques

## Abstract

Magnetic skyrmions are topological magnetic spin structures exhibiting particle-like behaviour. They are of strong interest from a fundamental viewpoint and for application, where they have potential to act as information carriers in future low-power computing technologies. Importantly, skyrmions have high physical stability because of topological protection. However, they have potential to deform according to their local energy environment. Here we demonstrate that, in regions of high exchange energy density, skyrmions may exhibit such extreme deformation that spontaneous merging with nearest neighbours or spawning new skyrmions is favoured to attain a lower energy state. Using transmission electron microscopy and a high-speed imaging detector, we observe dynamics involving distinct configurational states, in which transitions are accompanied by spontaneous creation or annihilation of skyrmions. These observations raise important questions regarding the limits of skyrmion stability and topological charge conservation, while also suggesting a means of control of skyrmion creation and annihilation.

## Abstract (web summary)

Skyrmions are spin textures which have integer topological charge. Typically they are very stable. Here, the authors observe the spontaneous creation and annihilation of skyrmions in regions of high exchange energy density, suggesting a limit to skyrmion stability.

## Introduction

Skyrmions were originally proposed as a soliton model of the nucleon by Tony Skyrme in 1962^1^, but they have since been observed as emergent topological quasiparticles in a variety of condensed matter systems, including superconductors^2^, liquid crystals^3^ and magnetic thin films^4^. Although the specific microscopic mechanisms leading to the formation of skyrmion structures differ in each case, the emergent topological structures bear many similarities and skyrmions are therefore of fundamental interest. Topology defines the distinctness of the geometries in continuous systems such as vector fields, as the inability to continuously map one system to another. Skyrmions can be understood as localised topological knots within continuous fields that cannot be unwound without a discontinuous break in their structure. This property is fundamental to the idea of topological protection, equipping skyrmions with a degree of stability against perturbations and contributing to the description of skyrmions as quasiparticles. Skyrmions can be categorised according to the topological charge defined by their structure, whereby processes in which they participate are expected to obey topological charge conservation. Understanding the real-world physical stability and possible interactions of these topological structures is of great importance, especially in magnetic thin films, in which magnetic skyrmions are expected to play a role in future low-power computing technologies^5^.

In non-centrosymmetric helimagnetic materials, such as B20 FeGe, the lack of inversion symmetry in the crystal structure, combined with strong spin–orbit coupling gives rise to competition between the Dzyalonshinskii–Moriya interaction (DMI) and the Heisenberg exchange interaction^6^. Owing to this competition, materials with a significant DMI will host a helical magnetic ground state, defined by continuously rotating magnetisation along a particular direction with an associated helical wavevector^7^. It is possible to describe the emergence of a hexagonal magnetic skyrmion lattice crystal (SkX) as a magnetic phase transition involving the nucleation of discrete topological quasiparticles from the helical ground state under favourable conditions of magnetic field and temperature^8^. The description of skyrmions as particle-like objects is supported by experimental observations that indicate their high stability^9^ and their ability to organise themselves into domains within a SkX^10^ (as commonly seen in atomic systems) or even microcrystals analogous to colloidal crystallisation^11^. In addition, the inherent rigidity of individual skyrmions allows current-induced propagation via spin-transfer torque without destruction^12^. It has been reported that the transition of a SkX into the topologically trivial ferromagnetic state (high field) or helical state (low field) involves magnetic singularities known as Bloch points that fulfil topological charge conservation during these transitions^13,14^. However, owing to the extreme difficulties associated with performing nanometre resolved magnetic imaging of a stochastic dynamic process taking place in the range of durations from nanoseconds to tens of microseconds, it remains to be experimentally determined whether topological charge is conserved during skyrmion annihilation processes.

Here, we report the direct observation of spontaneous, repeatable creation and annihilation of individual skyrmions in FeGe using Lorentz transmission electron microscopy and a high-speed imaging detector with a temporal resolution of 10 ms (see “Methods” for details). Creation and annihilation processes occuring on timescales of tens of milliseconds are observed as a repeated lateral skyrmion motion, captured (Supplementary Movie 1) across a SkX domain boundary separating lattice domains. These thermally driven transitions involve a number of configurational states with distinct lifetimes, separated by relative energy barriers and characterised by skyrmions of five- and sevenfold coordination (5–7 defects). Aided by micromagnetic simulations, we show that these transitions occur in regions of localised high-energy density, dominated by exchange energy, while the wider SkX remains in the stable ground state under conditions of constant applied field and temperature. Furthermore, the observed transitions involve discrete changes in topological charge and we propose that extreme deformation of key skyrmions leads to the emergence and subsequent destruction of antiskyrmions, fulfilling topological charge conservation. This demonstrates an example of magnetic strain-limited stability for skyrmions and suggests that topological protection may be overcome through the spontaneous emergence of topological structures that provide lower-energy pathways for skyrmion creation or annihilation.

## Results

### Identifying and characterising configurational states

**Fig. 1:**
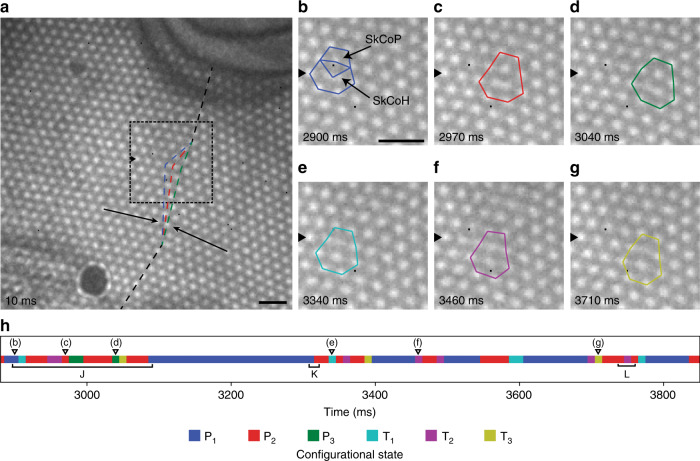
Fresnel TEM images of SkX and identification of configurational states. **a** Entire region of observed skyrmion lattice, showing the domain boundary with an average misorientation angle of 14° (black dashed line) and principle lattice vectors (arrows). The three coloured (blue, red and green) dashed lines demonstrate how the domain boundary shifts during transitions between the P_x_ states and the associated lateral skyrmion motion. Scale bar, 200 nm. **b**–**g** Examples of the six configurational states observed, with the time of each frame given in the bottom left corner of each image. The black arrowheads point along the row of skyrmions, in which lateral motion was taking place (same position in each image). Skyrmions with seven- and fivefold coordination (SkCoH and SkCoP, respectively) are highlighted by the conjoined pentagons and heptagons. Scale bar, 200 nm. **h** A portion of the transition data showing the progression of reversible transitions between states shown in **b**–**g**. The markers (**b**–**g**) additionally indicate the time of occurrence of the images shown in **b**–**g**. Colour in each panel is associated with the identified configurational states, according to the bottom key.

Transitions between six unique configurational states (Fig. 1b–g) were observed within the region defined by the dashed box in Fig. 1a. These states are further categorised into three primary states, namely, P_1_, P_2_ and P_3_ (Fig. 1b–d), and three transition states, namely, T_1_, T_2_ and T_3_ (Fig. 1e–g). Each of the identified states features a lattice defect structure composed of skyrmions of five- and sevenfold coordination (5–7 defects), highlighted in Fig. 1b by the conjoined pentagon and heptagon, structurally analogous to the 5–7 defects found in graphene^15^. Note, only the overall outline of the 5–7 defect is shown in later images. The skyrmions at the centre of the pentagons and heptagons, herein denoted as SkCoP and SkCoH, respectively, differ structurally from regular lattice skyrmions which possess hexagonal symmetry^16^ by being more spatially compressed (under fivefold coordination) or expanded (under sevenfold coordination)^17^. The motion of the 5–7 defects changes the position of the domain boundary (shown by the blue/red/green dotted lines in Fig. 1a) and is accompanied by very slight shifts in the regular lattice skyrmions in the immediate vicinity as they accommodate the change.

**Fig. 2:**
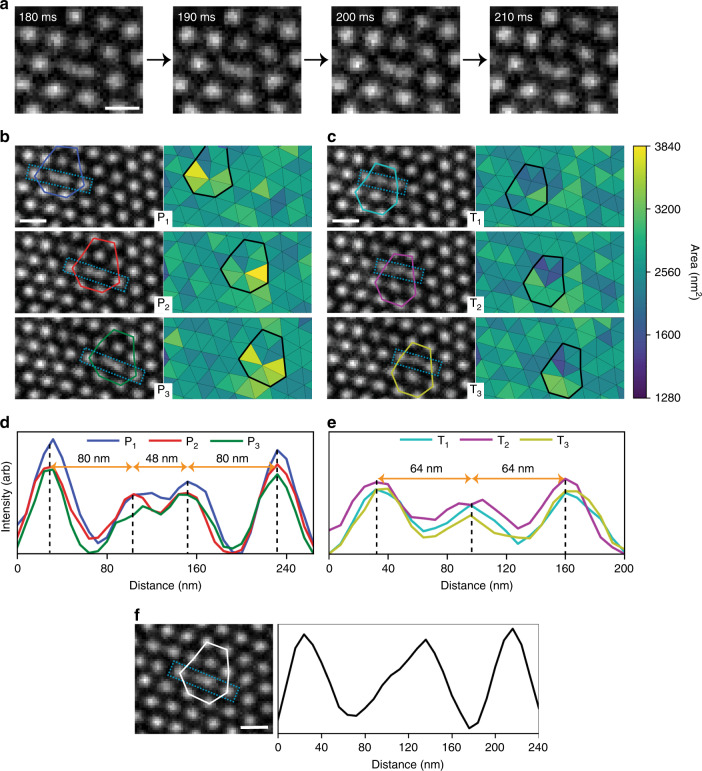
Spatial analysis of the six observed configurational states. **a** Successive frames showing the variation in structure of the SkCoH in the P_1_ state with the time of each frame given in the top left of each image. Scale bar, 100 nm. **b**, **c** Summed intensity images of each observed state with coloured outlines (same colour key introduced in Fig. 1) around the 5–7 defects (left) and their Delaunay triangulation calculated from the skyrmion geometric centre of mass (right). The colour bar indicates the area of the triangles in nm^2^. Scale bars, 100 nm. **d**, **e** Integrated line-profile plots of the P_x_ and T_x_ states, respectively, measured across the regions shown in **b**, **c**, (dashed rectangles). The two outermost peaks are associated with skyrmions of regular sixfold coordination. The double inner peak in **d** demonstrates the dumbbell-like structure of each of the SkCoH in the P_x_ states. **f** Summed intensity image and integrated line-profile plot across a stationary (under observation) SkCoH, for comparison. This stable 5–7 defect is located further down the SkX boundary, just below the right black arrow in Fig. 1a. Scale bar, 100 nm.

In order to analyse the order of occurrence, each frame was assigned to one of the observed P_x_ or T_x_ states (see “Methods”). A portion of this sequence data can be seen in Fig. 1h. The system was observed to transition reversibly between P_x_ states via T_x_ states, an example of the reversibility of the transitions is recorded in section J of Fig. 1h. However, some P_x_–P_x_ transitions observed did not visibly appear to proceed via a T_x_ state (see section K in Fig. 1h). In these cases, we assume that, owing to its short-lived nature compared with the frame-acquisition time, occupation of the T_x_ state simply comprised a small fraction of the duration of an individual frame. Evidence for such an assumption comes from observations in which some smearing of intensity suggested multiple states (P_x_ and T_x_) being captured in a single frame and will be discussed further below. Furthermore, the system did not always transition between different P_x_ states, as recorded in section L of Fig. 1h. This pattern suggests that, once the system is in a T_x_ state it can lower its energy by transitioning to a neighbouring P_x_ state or back to its starting P_x_ state. In addition, the transition probabilities (Supplementary Fig. 1) demonstrate that the system does not randomly transition between the six states, but rather hops between adjacent P_x_ states via the appropriate T_x_ state. Finally, we describe these transitions in terms of skyrmion creation and annihilation. During P_x_ to T_x_ transitions, a SkCoP is created, appearing to be formed by the division of an expanded skyrmion of sevenfold coordination, SkCoH. Conversely, in a T_x_ to P_x_ transition, a skyrmion of fivefold coordination, SkCoP, is annihilated by merging with a nearest-neighbour skyrmion. Thus, skyrmion creation is associated with P_x_ to T_x_ transitions, and skyrmion annihilation is associated with T_x_ to P_x_ transitions. In T_x_ states, there is higher local skyrmion density than in P_x_ states, which, through there being a reduction of the mean inter-skyrmion distance, we infer there to be an increase in local energy density. The precise structure of the skyrmions within the 5–7 defects is elucidated through the spatial analysis of the six observed states. A series of four successive frames of the P_1_ state (Fig. 2a) shows that the SkCoH appears to have distinct curvature around its centre and that this curvature increases as the sequence progresses. This behaviour demonstrates the dynamic nature of the SkCoH structure, in which the skyrmion appears to be deforming between elongated and dumbbell structures. Such structures represent a significant deformation from the regular hexagonally coordinated lattice skyrmions. Each pair of images in Fig. 2b, c, consists of a summed intensity image across all observed examples of the state (left) and a Delaunay triangulation analysis of the average skyrmion positions based on their geometric centre of mass (right). In Fig. 2d, e, integrated intensity profiles (extracted from the dotted box in each summed intensity image in Fig. 2b, c) are plotted. They record the intensity values along a path joining two skyrmions of regular sixfold coordination, passing through a SkCoH in the case of P_x_ states (Fig. 2d) or through a SkCoP in the case of T_x_ states (Fig. 2e).

A key feature of the P_x_ states is the relatively deformed structure of the SkCoH in the 5–7 defects (Fig. 2b). From the summed intensity images, the deformed structure appears to possess the aforementioned dumbbell shape on average. This observation is supported by the intensity profiles in Fig. 2d, which show additionally that the SkCoH in each of the P_x_ states has a highly similar form. The intensity profiles are characterised by two clearly defined central intensity peaks of approximately equal height, with an intensity 60–70% that that of regular neighbouring skyrmions of sixfold coordination, and spatial extent double that of regular neighbouring skyrmions of sixfold coordination. Figure 2d also shows that the same-row nearest-neighbour distances (i.e., from the intensity peaks of the SkCoH dumbbell to the intensity peaks of the adjacent regular hexagonally coordinated skyrmions) approximately match those of the bulk lattice: 80 nm. By comparing the SkCoH structure of the two most common states (P_1_ and P_2_), it was observed that 41% (205 of 499 frames showing P_1_) and 87% (328 of 376 frames showing P_2_) were characterised by a SkCoH skyrmion possessing an intensity central double peak, as described for the summed intensity images in Fig. 2b. This observation suggests that the precise structure of the SkCoH fluctuates, but a common state (indeed the most common state for the less stable P_2_ state) is the dumbbell-like form.

Following a similar set of analyses for the T_x_ states, the SkCoP in the 5–7 defects appears to be less well defined in the summed intensity images appearing somewhat smeared (Fig. 2c). The intensity profiles in Fig. 2e across the three skyrmions involved show three defined intensity peaks, with a central peak intensity ~75–90% that of the neighbouring peaks. Between their positions, the intensity decreases to ~58–68% of the central peak values, highlighting the transient nature of the T_x_ states. The compressed nature of the SkCoP in the T_x_ state can also be observed in both the Delaunay triangulation map of Fig. 2c and in the intensity profile of Fig. 2e, in which same-row nearest-neighbour distance is measured to be 64 nm, 20% closer than in the bulk lattice. This deviation from the equilibrium inter-skyrmion distance corresponds to an increase in energy density (see Supplementary Fig. 2b).

A non-time varying 5–7 defect (from a different location along the same SkX domain boundary) is shown in Fig. 2f, with the intensity across the SkCoH shown in the integrated line profile. Again, the SkCoH is more spatially extended than regular hexagonally coordinated lattice skyrmions, but, instead of the two distinct central intensity peaks that were observed for the dynamic SkCoHs in Fig. 2b, there is a single peak with a shoulder. This pattern indicates that this stationary SkCoH more likely possesses a single core, displaced towards one side. The distance between the two intensity peaks belonging to regular skyrmions in Fig. 2f is ~192 nm, whereas this same distance is ~208 nm in the P_x_ states in Fig. 2d. This indicates that the SkCoHs involved in 5–7 defects dynamics have a greater lateral spatial extent, giving rise to the additional deformation observed that yields the dumbbell structure. This behaviour may be a contributing factor for the skyrmion creation process through modification of the energy landscape. Comparing the dynamic and stationary SkCoH integrated intensity line profiles (Fig. 2d, f, respectively), the lower relative central peak intensity and greater spatial extent suggests higher variance in the dynamic SkCoH structure. Fresnel (defocused) Lorentz transmission electron microscope (TEM) imaging, while providing high-contrast imaging of the skyrmions, does not possess sufficient resolution to allow the identification of the detailed magnetic structure within the deformed skyrmions^8^. However, it is possible to correlate the Fresnel images with higher-resolution differential phase-contrast images of 5–7 defects, as will be discussed presently.

By comparing the Delaunay triangulation images in Fig. 2b, c, it is clear that the triangular cells joining each skyrmion location have a lower area in the T_x_ states, making the overall nearest-neighbour distances shorter than in the P_x_ states (shown by the colour scale). This difference reinforces the fact that skyrmion creation causes regions of higher skyrmion density (T_x_ states) that reduce local energetic stability, leading to later relaxation to a P_x_ state through annihilation of a skyrmion. The fact that these transitions occur spontaneously suggests that the relative energies of the six states are close and that the thermal energy fluctuations of the system are sufficient to overcome the potential energy barriers separating them.

### Relative stability and energy landscape

In order to investigate the energetics of the system, the lifetimes of each state over the 1000 frames were measured.

**Fig. 3:**
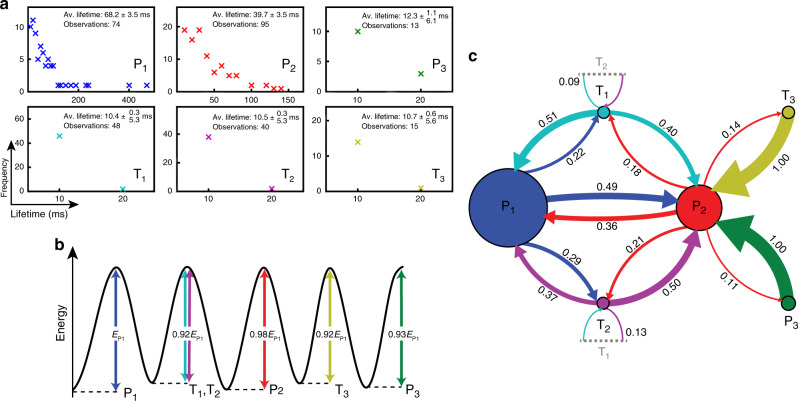
Lifetime distributions, associated energy barriers and transition probabilities of each state. **a** Scatter plots of lifetime distributions for each of the observed configurational states. Half the frame time (5 ms) was added to the lower bound in T_x_ and P_3_ lifetime uncertainties due to the short-lived nature of these states not always being captured individually per frame. **b** Relative energy barriers calculated using the Néel–Arrhenius equation and the average lifetime associated with each state. Each barrier height is given relative to the most stable state (P_1_), which had an energy barrier height on the order of 0.55 ev. **c** Observed state transitions represented as a Markov chain process, only including transitions observed more than once. Circles (arrows) representing each state (transition) are scaled to the relative lifetimes (transition probabilities).

Figure 3a shows the lifetime distributions along with the total number of observations of each of the six states, indicating that the P_1_/T_1_ and P_2_/T_2_ states were the most commonly observed. Owing to the short-lived nature of the T_x_ and P_3_ states, these transitions may not always be captured individually, but may instead be averaged across the frame duration as previously mentioned. This explanation also suggests why T_x_ states were not always observed during transitions, since (for example) state lifetimes of 1 ms would contribute only 10% intensity to the final 10 ms frame. It has been established that the system can transition to or from the same P_x_ state (P_1_–T_1_–P_1_ for example). However, if the T_x_ state was very short-lived, then it is possible that the system could appear to have stayed in the P_x_ state (P_1_–P_1_). This summing effect might explain some observations of very long lifetimes (~400 ms) for the P_1_ state in Fig. 3a.

The timescale of a magnetic transition between two states, facilitated by the thermal energy of the system, while separated by a simple potential energy barrier, can be deduced from the Néel–Arrhenius equation:

1 $$\frac{1}{\tau } = {\upnu}_0(T){\mathrm{e}}^{ - \frac{{\Delta E}}{{kT}}},$$

where the lifetime *τ* of a particular state is the product of the attempt frequency *ν*_*0*_(*T*) (number of transition attempts per second) and an exponential term containing the Boltzmann factor, which gives the probability of a transition with energy barrier Δ*E* at temperature *T*. By assuming that the observed transitions obey Eq. (1) to a first approximation and using an attempt frequency of 10^9^ Hz and *kT* of 0.022 eV (see “Methods” for details), relative energy barriers for the skyrmion creation and annihilation processes were calculated (Fig. 3b). The most energetically stable configuration is the P_1_ state (with a barrier height on the order of 0.55 eV), corresponding to its relatively long lifetime. The less stable P_2_ state has 28% more observations than P_1_, this disparity can be understood by the fact that the P_2_ state is both energetically and spatially positioned between the P_1_ and P_3_ states. This suggests that there may be entropy-compensation effects involved, due to the increased number of possible transition state configurations accessible from the P_2_ state (decreasing its observed lifetime)^18,19^.

By assuming that each transition is not affected by past transitions, the transition behaviour can be modelled using a Markov chain. The observed transition probabilities and average lifetimes of each state were combined to give the schematic in Fig. 3c. In this diagram, only transitions which occurred more than once are included and the arrows (circles) are scaled to the relative transition probabilities (lifetimes). During a transition from P_1_ to P_3_, it is more probable for the system to proceed in multiple lower-energy steps via the P_2_ state instead of a single step. This multistep process is demonstrated by the individual transition probabilities given in Fig. 3c, where zero transitions were observed between the P_1_ and T_3_/P_3_ states directly (see Supplementary Fig. 1 for the full transition matrix). Instead, every state has a connection both to and from the P_2_ state (red circle). It should be noted that the T_1_ and T_2_ states are labelled at the same position in the energy landscape (Fig. 3b). This is because although they are spatially distinct (Fig. 2c), they differ only by a small shift in the skyrmion positions local to the SkCoP and therefore were approximately equally likely to occur. However, Fig. 3c demonstrates that there is a small probability bias when transitioning from a T_x_ state to the corresponding P_x_ state. For example, since T_2_ to P_2_ requires a smaller overall shift in skyrmion positions it is more probable than T_2_ to P_1_.

### Skyrmion creation and annihilation mechanisms

In order to gain insight into the energy terms that might govern the skyrmion creation and annihilation processes, we performed a series of micromagnetic simulations of both isolated 5–7 defects and those within a SkX boundary (see Supplementary Note 1). We found that the stretched core of the SkCoH is a region of high-energy density due to an extended region of magnetisation antiparallel to the applied perpendicular magnetic field (providing a large positive Zeeman energy contribution). The magnitude of this increase in energy was found to be determined by the degree of deformation (spatial extent) of the SkCoH. Regions of increased exchange energy density around the SkCoP were observed due to the reduction from the equilibrium of the inter-skyrmion distance. Thus, it follows that the splitting of an expanded SkCoH (P_x_ to T_x_) lowers the Zeeman energy but in creating an extra skyrmion, leads to regions of high skyrmion density (T_x_ states) with steeper magnetisation gradients, increasing the local exchange energy. Therefore, these states quickly collapse back into one of the P_x_ states via skyrmion annihilation (T_x_ to P_x_), lowering the skyrmion density, but resulting in significantly deformed SkCoHs with a high Zeeman energy contribution. We propose that the interplay and balance of these two energy configurations results in the observed repeated, spontaneous transitions involving skyrmion creation and annihilation. Due to limitations in the temporal resolution of the LTEM measurements, the fine details of the transition mechanisms are not yet accessible experimentally. However, informed by micromagnetic simulations (see “Methods” and Supplementary Note 2 for details), we propose mechanistic pathways involving antiskyrmions for both creation and annihilation of skyrmions through splitting and merging processes.

**Fig. 4:**
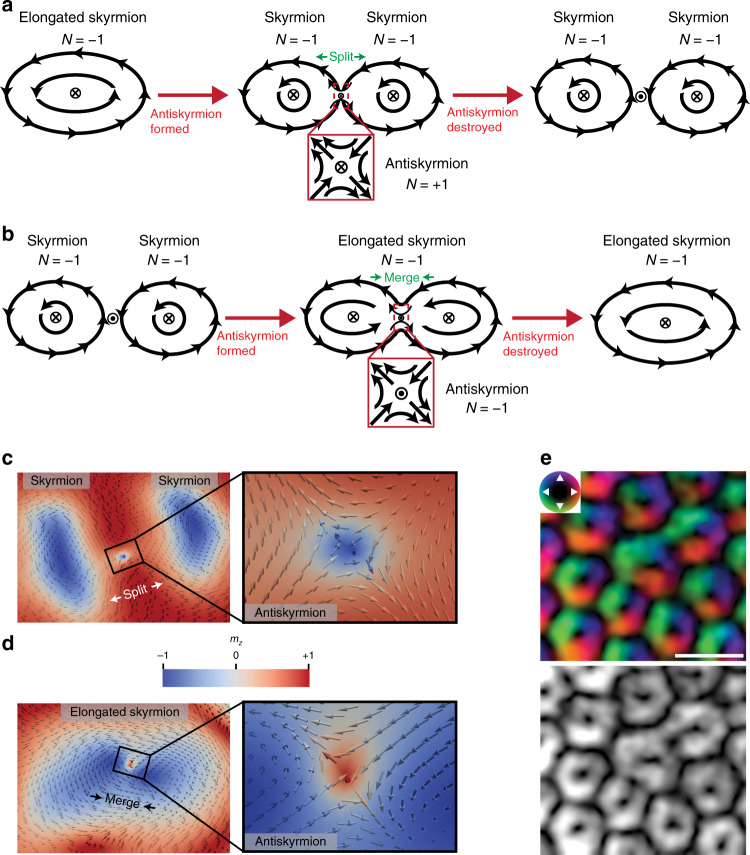
Skyrmion creation and annihilation mechanisms and investigation of the deformed SkCoH structure. **a**, **b** Proposed mechanisms for skyrmion creation and annihilation via splitting and merging, respectively. *N* gives the topological charge of each structure involved. **c**, **d** Micromagnetic simulation snapshots of splitting and merging transition states, closely resembling the middle states in **a**, **b**, respectively. Colour bar indicates the out-of-plane (*m*_z_) component of magnetisation. **e** DPC images of a stationary 5–7 defect showing pronounced deformation of the SkCoH structure and indication of merging with nearest-neighbour skyrmions. Colour (top) is given by the inset colour wheel, which indicates the direction of the in-plane magnetisation, where black indicates out-of-plane magnetisation. Scale bar, 100 nm.

In Fig. 4, we take account of the topological charges associated with skyrmions and suggest mechanisms that explain how our experimentally observed processes may be accomplished. Figure 4a shows how a significantly deformed SkCoH with a topological charge of *N* = −1 involved in the observed dynamic processes may split into two separate skyrmions (P_x_ to T_x_ transition). This first occurs through stretching of its form from an elongated structure to a dumbbell structure (as shown experimentally in Fig. 2a). Such a deformation is continuous, and consequently, there is no associated change in topology or topological charge. However, at the centre of the dumbbell-like SkCoH structure, significant magnetisation gradients and curvature occur, creating a region of locally high-energy density and low energetic stability. This leads to formation of two skyrmion structures with a total topological charge of *N* = −2, connected by a central region containing an antiskyrmion with *N* = +1 (positive topological charge since it has the same core polarity as the two formed skyrmions). Thus, the antiskyrmion fulfils topological charge conservation during the SkCoH splitting into two skyrmions. Since the middle state in Fig. 4a could not be captured experimentally, we investigated its creation using micromagnetic simulations, through enforced splitting of an elongated skyrmion using magnetic field gradients (Supplementary Fig. 3e). The resulting transition state can be seen in Fig. 4c, where two individual skyrmions are joined by an antiskyrmion. The final step involves the unstable antiskyrmion reducing in size, until eventually the central spin rotates in-plane and the antiskyrmion topology is destroyed, accompanied by a step change in total topological charge of *N* = −1 to *N* = −2 (Supplementary Fig. 3b, right panel). This antiskyrmion collapse mechanism has been proposed theoretically by Desplat et al.^19^, where the saddle point configuration corresponds to the state preceding the central spin flipping (Fig. 4c, right panel).

Figure 4b demonstrates the case for skyrmion merging (T_x_ to P_x_ transition). We propose that the reverse mechanism is followed such that a reduction in proximity of two merging skyrmions with a total topological charge of *N* = −2 leads to a singled elongated skyrmion with *N* = −1, with a trapped region of magnetisation parallel to the external applied field. This is an antiskyrmion of opposite polarity to the previous splitting case (Fig. 4a) and thus has an associated topological charge of *N* = −1. Therefore, the antiskyrmion again fulfils the role of topological charge conservation during skyrmion merging. Support for such a mechanism again came from micromagnetic simulations where applied magnetic field gradients caused merging of two neighbouring skyrmions (see Supplementary Fig. 3d). The resulting transition state and antiskyrmion corresponding to the middle state in Fig. 4b can be seen in Fig. 4d. Again, the antiskyrmion reduces in size until it is eventually destroyed when the central spin flips, resulting in a step change in topological charge from *N* = −2 to *N* = −1 (Supplementary Fig. 3b, left panel). The reverse mechanism is similar to one suggested for skyrmion lattice inversion during polarity switching of an applied external magnetic field, whereby antiskyrmions are formed at the point of skyrmion core merging^20^.

This notion of antiskyrmions conserving topological charge during creation or annihilation events has also been studied in dipolar magnets, in which antiskyrmions have a higher stability than that in chiral magnets^21^. The thickness of our sample (on the order of the FeGe helical length, 70 nm) implies that variation in magnetisation through the thickness will be minimal. This leads us to believe that the mechanism involves the creation and annihilation of antiskyrmions rather than the involvement of Bloch point zippers, as discussed in ref. ^13^, which studied a bulk sample. However, both of these mechanisms involve topological structures that are created and eventually destroyed to fulfil topological charge conservation during transitions. The mechanisms involving antiskyrmions demonstrated in Fig. 4 also bear resemblance to the proposed mechanisms of magnetic vortex core reversal, whereby antivorticies are created and subsequently destroyed^22^.

Recently, a theoretical study using a saddle point search method was used to determine saddle point configurations of skyrmions undergoing duplication and collapse^23^. These configurations bear many similarities with the dumbbell-like structures observed and are suggestive of the proposed transition state configurations involving antiskyrmions. It has been shown that, using a 360° domain wall model, skyrmion deformation does not significantly lower the universal skyrmion energy (i.e., energy required to reduce skyrmion radius to zero)^24^. This value should be sufficient to prevent spontaneous skyrmion annihilation processes at the temperatures studied here. However, we propose that thermal fluctuations combined with extreme skyrmion deformation lead to the formation of the antiskyrmions as described. Thereby providing new energy pathways for skyrmion creation and annihilation that are much lower than the calculated universal skyrmion energy.

To better understand the magnetisation distribution within deformed SkCoH/SkCoP, we performed comparisons with other 5–7 defects not varying in time (i.e., stationary under observation). Figure 4e shows a static 5–7 defect from a different SkX imaged using the higher spatial resolution technique of differential phase contrast from four-dimensional scanning transmission electron microscopy (4D STEM) (see “Methods” for details). The images show that the SkCoH is quite significantly deformed compared with the lattice skyrmions surrounding it. For the magnitude of the in-plane component of magnetic induction (Fig. 4e, bottom panel), the SkCoH exhibits a significant deformation with clearly defined strip-like regions of in-plane magnetisation connecting it to two neighbouring skyrmions, isolating out-of-plane regions as suggested in Fig. 4b. In addition, it is possible to identify a stretched core region and some kinking of the magnetisation circulating around it. This situation bears similarity to the structure suggested by the Fresnel images of the SkCoH seen for the dynamic 5–7 defects (Fig. 2), but does not possess the deformation required for splitting at the temperatures studied.

## Discussion

We observed spontaneous dynamic processes involving the creation and annihilation of individual skyrmions at SkX domain boundaries. Frame-based analysis allowed us to identify key states in the process and to observe variations in the structure of the SkCoH/SkCoP in 5–7 defects that leads to skyrmion creation or annihilation. Naturally, the processes observed are governed by thermal fluctuations and induced exploration of a relative potential energy landscape and appeared at first sight not to fulfil the concept of topological charge conservation. However, aided by micromagnetic simulations and based on our high-spatial-resolution imaging of other deformed but time-non-varying SkCoH skyrmions, we have proposed that skyrmions can exhibit extreme deformation, leading to the formation of antiskyrmions. Through subsequent random local magnetisation fluctuations, the antiskyrmion object may be destroyed, and subsequently lead to lower-energy pathways involving neighbouring skyrmions merging or separating.

Our observations provide evidence of magnetic strain-limited skyrmion energetic stability in lattices, i.e., based on Zeeman and exchange interaction energies. This finding is contrary to many reports of high energetic stability, arising from topological protection for both isolated skyrmions and those in lattices^24–27^. Our observations provide the potential for a new method of controlling skyrmion creation and annihilation through engineering SkX boundaries with high magnetic strain, induced via the intentional patterning of magnetic/non-magnetic defects with key dimensions comparable to SkX periodicity. Although this study was limited to investigating a single SkX boundary, we have generally observed that 5–7 defects density and the rate of skyrmion dynamic events increase as a function of SkX misorientation angle^28^. Further time-resolved imaging studies of the extremely deformed skyrmions involved in these dynamic processes may provide new insights into the dynamic transformation processes of topological structures within condensed matter systems.

## Methods

### Lorentz-Fresnel TEM imaging

Sections of single-crystal FeGe were extracted from a bulk host crystal using the in situ liftout technique in a focused ion beam-scanning electron microscope (FIB-SEM) instrument (FEI Nova 200 Nanolab). The extracted sections possessed a (110) normal and thickness <100 nm in order to provide sufficient electron transparency. In the TEM (JEOL ARM200cF), the sample was cooled using liquid nitrogen in a Gatan HC3500 specimen holder. For Fresnel mode, defocused imaging of the skyrmion state was performed at a temperature of 253 K with the TEM operating in Low Mag (objective lens OFF mode). Out-of-plane magnetic fields were applied by partially exciting the objective lens. States containing multiple Skyrmion crystal lattices, and therefore boundaries, were induced by starting from a disordered helical state and then by applying and continually increasing the strength of the applied magnetic field up to a value of 510 Oe. Direct filming of the dynamics occurring at skyrmion lattice boundaries was performed using a high-speed, direct illumination, single-electron counting 256 × 256 pixel imaging detector (Quantum Detectors Merlin for EM 1R). In these experiments, filming was performed using an exposure time of 10 ms and in continuous read/write mode (i.e., no time gaps between frames), yielding a frame rate of 100 fps.

### Lorentz DPC imaging

DPC imaging of skyrmions, stationary under observation, was performed also on a JEOL ARM200cF operating in a custom LM-STEM mode^29^. An electron probe with semi-convergence angle 415 mrad and full-width-half-maximum of 6 nm was scanned across the sample. 4D STEM acquisition was performed over a 256 × 256 pixel scan array, 1 ms pixel dwell time (using the same pixelated single-electron counting detector as for the Fresnel imaging) to acquire a dataset that was subsequently processed using DPC phase-correlation algorithm in the fpd library^30^. Isotropic smoothing was applied to the resultant image in order to reduce noise effects and to make clear the features of the distorted skyrmion structure.

### Image analysis

Assignment of each image frame to one of the configurational states was performed by first using the centre of mass of intensity of the skyrmions involved in the lateral motion to create Delaunay triangulation maps. Then, visual inspection and integrated intensity line-profile measurements allowed the identification of the six unique observed states. A Delaunay triangulation of each individual frame was then categorised into one of the six observed states. All analysis of Fresnel images shown in Fig. 2 first involved a background subtraction using the rolling ball algorithm. The images shown in Fig. 2 are the result of the summation of many frames in order to significantly reduce influence from the level of statistical noise associated with the limited electron counts in the short movie frames.

### Assumptions for relative energy barrier calculations

A precise value of the temperature-dependent attempt frequency *ν*_*0*_(*T*) in Eq. (1) can be calculated only from detailed information about the minimum energy path taken during transitions^31^, the exact mechanism of which has not yet been determined for skyrmion merging and splitting. However, it has been reported that the attempt frequency for magnetic skyrmion annihilation in thin films is ~10^9^–10^10^ Hz^32^, which is in the range typically reported for other magnetic systems^33^. Theoretical studies into the minimum energy pathway of isolated skyrmion collapse also report strong entropy-compensation effects under certain conditions, particularly metastable skyrmions far from equilibrium conditions leading to variations in *ν*_*0*_(*T*) of up to five orders of magnitude^19,34^. However, we justify the use of an attempt frequency of 10^9^ Hz since we are only seeking to calculate relative barrier heights. In addition, in this study we do not have experimental indication of the appropriate modification to *ν*_*0*_(*T*). Finally, any error in the estimated value of *ν*_*0*_(*T*) will simply shift the entire energy landscape vertically, leaving the relative barrier heights unchanged.

### Micromagnetic simulations

Micromagnetic simulations were performed using a finite difference (FD) approach, utilising the well-established MuMax3 code^35^. Material parameters were chosen in order to represent the estimated material parameters of FeGe^36^, they were *D* = 1.58 mJ m^−2^, *A* = 8.78 pJ m^−1^ and *M*_s_ = 384 kA m^−1^. Further details on simulation parameters can be found in Supplementary Notes 1 and 2. The purposes of the simulations were firstly to estimate the main energy contributions involved in 5–7 defect structures and secondly to gain insight into the possible mechanistic pathways of skyrmion splitting and merging. Therefore, we chose to perform all simulations at zero temperature, as we were not seeking to reproduce the dynamics observed experimentally. In order to enforce splitting and merging of individual skyrmions, a strong localised magnetic field gradient was imposed. This field gradient acted as a proxy for the magnetic strain across a boundary, forcing skyrmions to merge or split as observed experimentally. Therefore, the results may only give approximations to the true pathway. We chose ultra-thin films at zero temperature in order for the simulations to be valid, given the thickness-temperature-field phase space of B20 FeGe^20^. Finally, the micromagnetic simulations were performed to support insight into the mechanistic pathways which are currently inaccessible experimentally due to timescales.

## Supplementary information

Supplementary Information

Supplementary Movie 1

Peer Review File

Description of Additional Supplementary Files

## Data Availability

The Lorentz-Fresnel, DPC and micromagnetics data that support the findings of this study are available in the Enlighten: Research Data repository (10.5525/gla.researchdata.1030)^37^.
