# Supplementary material for: Spontaneous creation and annihilation dynamics and strain-limited stability of magnetic skyrmions
Source: Nat Commun. 2020 Jul 15;11:3536. doi: 10.1038/s41467-020-17338-7 (PMC7363836; doi:10.1038/s41467-020-17338-7)
Supplement: Supplementary file 1 — Supplementary Information [file 41467_2020_17338_MOESM1_ESM.pdf]

Spontaneous creation and annihilation dynamics and strain-limited stability of magnetic skyrmions

Rendell-Bhatti et al.

Supplementary Information

Supplementary Figure 1: Transition Matrix

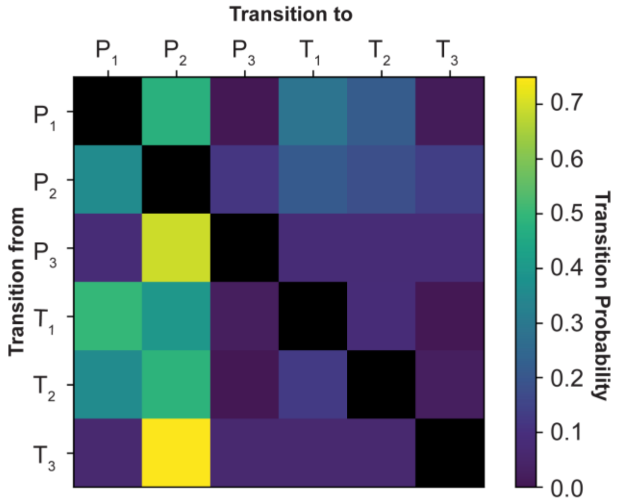

Supplementary Fig. 1 | Transition matrix of all observed transition probabilities. Transition matrix with probabilities normalised to unity across rows. This matrix highlights the involvement of the P_2 state with most transitions due to its position both geometrically and energetically in the observed transitions. Additionally, the P_3 and T_3 states transitioned to almost exclusively the P_2 state. Colour bar indicates the transition probability of each transition.

Supplementary Note 1: Micromagnetic simulations of skyrmion lattice boundary

Investigation of the relative energetic stability of 5–7 defects within a domain boundary was performed using finite difference micromagnetic simulations (MuMax3[1]) involving a pair of isolated 5-7 defects and those within a SkX domain boundary. In order to compare the relative energy densities of a 5-7 defect and regular 6-fold coordinated skyrmions, the appropriate equilibrium lattice parameter was found by varying the dimensions of a SkX triangular unit cell and minimising the energy with an external field of 0.2 T applied out-of-plane [2]. Supplementary Fig. 2a,b shows the relationship between total energy density, η_{tot}, and skyrmion lattice parameter, a_{sk}. The minimum in Supplementary Fig. 2a corresponds to the equilibrium skyrmion lattice parameter, approximately 91 nm, which was the lattice parameter used for the following simulations. The energy density plots in Supplementary Fig. 2c,d (averaged over thickness) were obtained by simulating a thin film of FeGe using periodic boundary conditions in the XY-direction. The simulation parameters for outputs in Supplementary Fig. 2 were B_z = 0.2 T and a damping parameter of α = 0.1 with material parameters D = 1.58 mJ m^{-2}, A = 8.78 pJ m^{-1} and M_s = 384 kA m^{-1} in order to represent the material parameters of FeGe[3]. The initial simulation in Supplementary Fig. 2c (left) involves a repeating array of 64 skyrmions, with simulation dimensions 632 nm x 730 nm x 50 nm and a cell size of 1 nm^3. Subsequently, a single skyrmion was deleted using a strong localised field and allowed to relax into the state shown on the right. The SkCoHs show up clearly as regions of high energy density due to their elongated forms.

In order to achieve the bi-domain state shown in Supplementary Fig. 2d, the system was initialised in a randomly magnetised state and allowed to relax into a disordered helical state at zero applied magnetic field. A perpendicular magnetic field of 0.4 T was subsequently applied in order to nucleate skyrmions and allowed to evolve for a total simulation time of 1 µs. This length of time was required for the randomly distributed skyrmions to form a skyrmion lattice. This resulted in a multi-domain skyrmion lattice. However, the skyrmions were extremely confined and did not exhibit the hexagonal symmetry observed experimentally,[4] or the equilibrium lattice parameter calculated in Supplementary Fig. 2a. At this point, the perpendicular anisotropy constant was removed, the external field was reduced to 0.2 T. Simulation dimensions of 1670 nm x 980 x 50 nm, corresponded to a minimum energy density and an equilibrium skyrmion lattice parameter of approximately 91 nm. As with the isolated 5-7 defects, the SkCoHs within the boundaries in Fig. S2d can be identified through their elongated cores, corresponding to high total energy density. Additionally, the DMI+exchange energy density plot highlights the increased energy density associated with the compressed SkCoP due to deviation from the equilibrium skyrmion lattice parameter. Exchange and DMI are combined here, because of how MuMax3 calculates the exchange energies.

The relative energies of 5-7 defects when compared to regular 6-fold coordinated skyrmions was calculated for both the simulations shown in Supplementary Fig. 2c,d. The integrated energy was found within the regions enclosed by the dashed white lines and then divided by the total simulation volume enclosed by these regions. The energy density of 5-7 pairs calculated here vary between 0.4% and 0.7% less energetically stable than the 6-fold coordinated skyrmions. The difference is due to the energy associated with the SkCoH core, in Supplementary Fig. 2c they are more deformed (approximately 6% higher *XY*-spatial extent) relative to the regular skyrmions, and thus have a higher maximum energy associated with them (see the η_{tot} energy density scales).

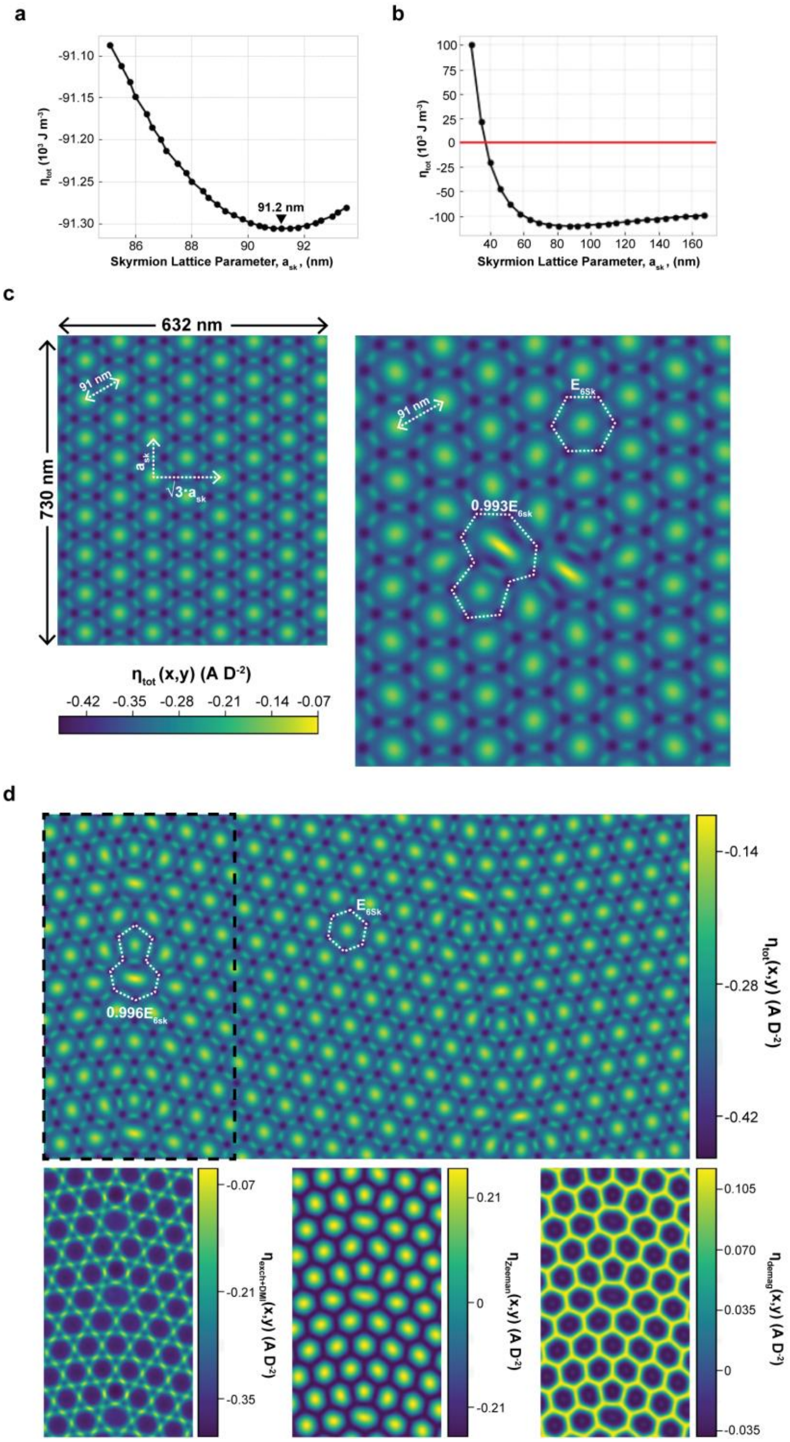

Supplementary Fig. 2 | Micromagnetic simulations involving 5-7 defects. a,**b**, Total energy density plots as a function of skyrmion lattice parameter, a_{sk}, for a thickness of 50 nm. Equilibrium lattice parameter of 91 nm was used in future simulations. **c**, Total energy density of continuous SkX (left) and with a pair of 5-7 defects imposed through skyrmion deletion (right). Energy density comparison of a 5-7 defect with regular 6-fold coordinated skyrmion calculated from the regions enclosed by the dashed white lines. **d**, Total energy density of SkX region containing two boundaries with three 5-7 defects in each. Energy density comparison again from the regions enclosed by the dashed white lines. Exchange+DMI, Zeeman and demagnetising energy density plots are from the left-most SkX boundary, enclosed by the black dashed box. A 0.2 T out-of-plane field is applied in all cases and energy densities are given in units of A/D^2.

Supplementary Note 2: Micromagnetic simulations of skyrmion creation/annihilation mechanisms

By using spatially confined short-duration magnetic field gradients, it was possible to initiate the splitting of an elongated skyrmion (Supplementary Fig. 3a top panel) and the merging of two neighbouring skyrmions (Supplementary Fig. 3a bottom panel). Supplementary Fig. 3d,e shows a series of magnetisation snapshots of skyrmion annihilation and creation, respectively. The colour is given by Supplementary Fig. 3c, where in-plane magnetisation is given by the colour and out-of-plane magnetisation is given by the shade where white points out of the page and black points into the page. The simulation parameters were D = 1.58 mJ m^{-2}, A = 8.78 pJ m^{-1} and M_s = 384 kA m^{-1}. A damping parameter of of α = 0.02 was used in order to investigate the dynamical aspects of the simulation under study here. A cell volume of 1 nm^3 was used, and the sample dimensions were 484 nm × 560nm × 2 nm for the merging case and 512 nm × 256 nm × 2 nm for the splitting case. The merging process was also carried out with a 10 nm thick sample in order to investigate the details of antiskyrmion destruction, and is shown in Supplementary Fig. 3f.

The merging simulation is shown in Supplementary Fig. 3d, a very short-pulsed (0.7 ns) magnetic field gradient (25 MT m^{-1}) was localised around a skyrmion in a hexagonal lattice using a two-dimensional gaussian with a standard deviation of 50 nm in the x-direction and 35 nm in the y-direction (shown by the dashed ellipse in the left panel of Supplementary Fig. 3a). This caused the skyrmion localised under the field gradient to move towards a neighbouring skyrmion and eventually merge. The merging of the two skyrmions results in continuous rotation of magnetisation (black circular arrow) and gives the appearance of an elongated skyrmion with an antiskyrmion superimposed at the centre (the trapped out of plane region). This antiskyrmion subsequently reduces in size, as shown in the bottom set of panels in Supplementary Fig. 3d. Once the antiskyrmion cannot be reduced in size any further, the central spin rotates in plane and the antiskyrmion topology is destroyed (this can be seen clearly in the second panel of Supplementary Fig. 3f). This is accompanied by a large discontinuous change in the topological charge (left panel in Supplementary Fig. 3b) which accounts for the removal of the N = -1 associated with the antiskyrmion. The splitting mechanism is shown in Supplementary Fig. 3e, again a short-pulsed (0.15 ns) magnetic field gradient was localised around the centre of an elongated skyrmion. The standard deviation of the gaussian in this case was 25 nm in both the x- and y-direction. In this case, the elongated skyrmion quickly separates into two skyrmions each with N = -1 and a central antiskyrmion with N = +1 (since it has opposite polarity to the antiskyrmion involved in Supplementary Fig. 3d). This conserves the initial topological charge of N = -1 of the single skyrmion. As before, the antiskyrmion first reduces in size and is then destroyed when the central spin rotates in plane. This is accompanied by a discontinuous change in topological charge (right panel in Supplementary Fig. 3c) corresponding to the removal of the N = +1 associated with the antiskyrmion.

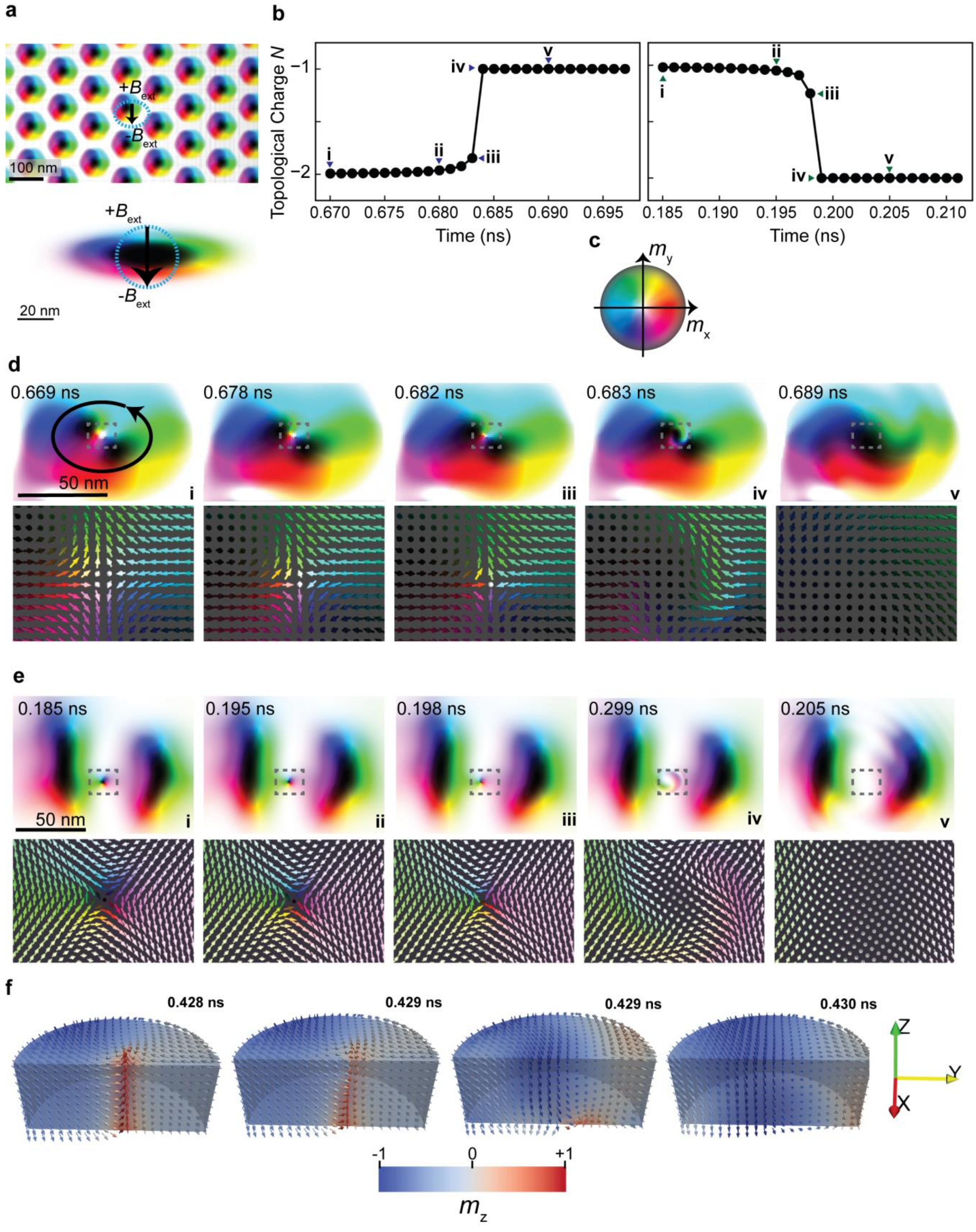

Supplementary Fig. 3 | Micromagnetic simulations of skyrmion creation/annihilation mechanisms. a, Initial states of skyrmion annihilation (top) and creation (bottom). Dashed ellipses give the first standard deviation of the field-pulse gaussians, with the arrow showing the direction of the magnetic field gradient from positive to negative field. **b**, Total topological charge as a function of time during the annihilation (left) and creation (right) simulations, labels i-v correspond to the panels in **d** and **e**. **d**,**e**, A series of scalar plot snapshots of magnetisation (top) and vector plots of the antiskyrmion (bottom) of the skyrmion annihilation/creation mechanisms respectively, colour given by **c**. **f**, Annihilation of an antiskyrmion through a thickness of 10 nm during the merging of two skyrmions.

1. Vansteenkiste, A. *et al.* The design and verification of MuMax3. *AIP Adv.* **4**, 107133 (2014).
2. Shibata, K. *et al.* Temperature and Magnetic Field Dependence of the Internal and Lattice Structures of Skyrmions by Off-Axis Electron Holography. *Phys. Rev. Lett.* **118**, (2017).
3. Beg, M. *et al.* Ground state search, hysteretic behaviour, and reversal mechanism of skyrmionic textures in confined helimagnetic nanostructures. *Sci. Rep.* **5**, (2015).
4. McGrouther, D. *et al.* Internal structure of hexagonal skyrmion lattices in cubic helimagnets. *New J. Phys.* **18**, (2016).
