# Supplementary material for: Spontaneous creation and annihilation dynamics and strain-limited stability of magnetic skyrmions
Source: Nat Commun. 2020 Jul 15;11:3536. doi: 10.1038/s41467-020-17338-7 (PMC7363836; doi:10.1038/s41467-020-17338-7)
Supplement: Supplementary file 4 — Description of Additional Supplementary Files [file 41467_2020_17338_MOESM4_ESM.pdf]

Title: Supplementary Movie 1

Description: Fresnel TEM video containing 1000 frames with a frame acquisition time of 10 ms and a total time of 10 s. Supplementary Movie 1 shows the spontaneous merging and separation of skyrmions across a skyrmion domain boundary.
