# Supplementary material for: Spontaneous creation and annihilation dynamics and strain-limited stability of magnetic skyrmions
Source: Nat Commun. 2020 Jul 15;11:3536. doi: 10.1038/s41467-020-17338-7 (PMC7363836; doi:10.1038/s41467-020-17338-7)
Supplement: Supplementary file 3 — Peer Review File [file 41467_2020_17338_MOESM3_ESM.pdf]

Reviewers' Comments:

Reviewer #1:
Remarks to the Author:
In their article „Spontaneous creation and annihilation dynamics and strain-limited of magnetic skyrmions" by F. Rendell-Bhatti and co-workers stability and transformations of skyrmions in the domain boundary region between two skyrmion grains in the prototypical skyrmion hosting B20 material FeGe is investigated by Lorentz transmission electron microscopy (LTEM).

The author focus on the continuous transformation of the position of individual skyrmion tubes in a particular 5-7 defect in the grain boundary. Here, the skyrmion arrangement is obviously not in its ground state and rearrangement of the skyrmion lattice is observed. Using a high speed detection system, the authors investigate the occurring configurational states, their spatial arrangement (also in comparison with a stable 5-7 defect), their statistics and their lifetime distribution. Using the simple Arrhenius law (and assuming that the attempt frequency is 10^9 Hz) the extract energy barriers for the spatial rearrangements. From their measurements and comparison to micromagnetic simulations they also infer a creation and annihilation mechanism which is reminiscent to the vortex core mechanism of magnetic vortices (not cited).

The paper is well written, and easy to follow. It relies on some assumptions (e.g. the attempt frequency mentioned above or the assumption that the short-lived Tx states can be somewhat captured with the temporal resolution available in the experiments).
However, I am very doubtful if an essentially 2D simulation of the skyrmion lattice is sufficient to capture the underlying physics here. Important, I guess, is the transition of the skyrmion tubes to new positions and this can initialize at various positions within the tube. What is more, the simulations are performed at zero temperature where the skyrmion tubes are static. In reality the wiggle around which can be seen in the experiments and motivates using the Arrhenius law. Without the comparison to micromagnetics an interpretation soley based on the LTEM images is hardly possible.
It is for this reason that I would like to ask the authors to consider more realistic simulations before resubmission to Nature Communications.

Reviewer #2:
Remarks to the Author:
This work is tackling the problem of topology concerning generation and annihilation of magnetic skyrmions which is of great interest in the field of magnetics. The authors investigated the dynamics of skyrmions at grain boundary of skyrmion crystals (SkX). They carried out the direct observation by taking Lorentz TEM movies of skyrmions in a thin plate of B20-type FeGe and extracted statistical information about dynamics of skyrmions through a detailed analysis on the Lorentz TEM movies. They also carried out micromagnetic simulations to support the observed dynamics of skyrmions. They demonstrated that the experimentally observed dynamics is governed by thermal activation in a system with energy landscape related to strain of magnetic structure. This finding provides an important clue to understand the structural stability and the topological aspect of skyrmions, and can be utilized for controlling skyrmions through strain of magnetic structure.

Overall, the experiments, analysis, and simulation are clear and of high quality, and the discussion provides detailed explanation and interesting insight. From physical point of view, however, there are some unclear points. Before a decision can be taken, I would like to give some comments to check scientific accuracy and (hopefully) improve the manuscript:

1) I feel Fig. 3b misleading because the colored broken lines are shown at the tops of the potential and not at the bottoms of the potential which is corresponding to the states.

2) The authors show asymmetric potential barriers in Fig. 3b. According to it, for example, the energy barrier from P1 to T1 is lower than that from T1 to P1. This should result in lower transition probability for transition from P1 to T1 than that for transition from T1 to P2. However, the authors

states at p.4, l.32 "Figure 3c also demonstrates the symmetry of the transitions, where transitions to or from a particular state had equal probabilities", which seems to contradict with the transition probabilities expected from the potential barriers. In my opinion, the symmetry of Fig. 3c is just due to appearance and due to the normalization. The authors extracted transition events so that total probability becomes 1. In the present figure, each row is multiplied by the frequency of each state. I suggest normalizing the probability for each row. In addition to it, I think the transition events can be modeled using Markov chain, and I also suggest providing the probability of self-transition, i.e. transition from a state to the same sate, as well.

3) In Fig. 4a and 4b, the authors show images of a stable 5-7 defect with explanation of "a static 5-7 defect from a different SkX". In Fig. 4c shows "A Fresnel image of a stable5-7 defect (from the same SkX domain boundary as the dynamic 5-7 defects)". However, the temperature and magnetic field is not shown explicitly for these images. Is the temperature and the magnetic field is the same as the other figures (253 K, 510 Oe)? If so, what is the difference of stable 5-7 defect and dynamic one?

4) In Fig. 4d and 4e, the authors show that the split and merge of skyrmions can be explained in terms of creation and annihilation of antiskyrmion localized at the middle of cores of the two skyrmions. This implies the energy barrier required for the transition takes place by two steps: first step is the deformation of skyrmions (the first red arrow) and the second step is the annihilation of antiskyrmion structure (the second red arrow). The first step seems to be clearly reasonable and to be achieved only by continuous deformation. The second step, however, the transition from the middle state of Fig. 4d and 4e to the last state is not discussed in the manuscript although it is not clear. Concerning this, I found that the only the difference between the middle state of Fig. 4d and 4e is direction of the magnetic moment of the antiskyrmion core. After that, I came to think the transition path from Px to Tx might be like this: Fig.4d initial state -(continuous deformation)-> Fig.4d middle state - (antiskyrmion core flip)-> Fig.4e middle state -(continuous deformation)-> Fig. 4e initial state. And, the transition path from Tx to Px is the opposite. However, I am confused because the flip of the magnetic moment at aitskyrmion core changes the topological chrage by 2, which cannot meet the consistency of the topological charge. Do the authors have any comment of my consideration or any clear answer about the second step?

5) I understand that even single temperature is enough for just comparing relative energy barrier scales if the observed events obeys the Neel-Arrhenius equation. However, this paper does not provide clear information that the observed events really obeys the Neel-Arrhenius equation. I think it would be better to include data at other several temperatures to support this if possible. Do the authors have any data to support or any comments about this suggestion?

6) The present analysis was done on the specific SkX grain boundary. In general, not limited to magnetic skyrmions, there are many types of grain boundaries of crystals due to many degrees of freedom, such as relative orientation of crystals and rigid body translation, and the stability and the properties differ depending on the grain boundaries. I think this also applies to two-dimensional SkXs. How can the present finding be generally applicable for the general grain boundaries of SkXs, for example, ones with a different misorientation angle?

7) Typo: an extra "regular" in caption of Fig. 2, l.6. "skyrmions of regular regular six-fold coordination".

Overall, the subject and experimental results of this work is exciting and has enough potential for publication in Nature Communications if the points discussed above have been dealt with correctly.

Reviewer #3:
Remarks to the Author:
In this study, the authors report the observation of spontaneous creation and annihilation of skyrmions in regions of high exchange energy density as the domain boundary of the skyrmion lattice in the FeGe thin film, using Lorentz TEM with a high-speed imaging detector, and Lorentz

DPC STEM. The distinct states are observed in dynamic 5–7 defect. The authors discuss that the spontaneous transition occurs via unstable antiskyrmions in the light of skyrmion stability and topological charge conservation. Although the transition is quite interesting and had not been performed experimentally in the thin films, I am hesitating to recommend publication of this manuscript in Nature Communications for the following reasons.

First, it is difficult to understand for me the intention of Figs. 4a-c (the DPC and Lorentz TEM images) to support the author's case as the main result where the creation and annihilation of skyrmions are mediated by antiskyrmions. The reason is that there is no clear description of the observed stationary states such as Px, Tx, intermediate state between Px and Tx. In addition, there is no description how the stationary images were experimentally obtained. Lower temperature than 253 K in Figs. 1, 2? Otherwise, if the stationary images were obtained at 253 K, the high-resolution DPC image makes no sense because of magnetically varying in time during scanning of electron beam.

Second, this study lacks the confirmed experimental evidence of the existence of both the elongated skyrmions (left in Fig. 4d and right in Fig. 4e) and the adjacent skyrmions completely divided into two (right in Fig. 4d and left in Fig. 4e) as the energy minimum states under thermal fluctuation. The reasons are as follows. As shown in attached Figs. 4a-c that add my comments, in the DPC images (Figs. 4a, b) the SkCoH is no longer single elongated skyrmion because of merging with the adjacent SkCoP and skyrmion. (As mentioned above, it is difficult to understand for me the intention of Figs. 4a-c.) On the other hand, all Lorentz TEM images cannot resolve the above merged state, as exemplified in Fig. 4c. Despite this Lorentz TEM resolution limit, the authors presuppose the existence of both the elongated skyrmions and the adjacent skyrmions, on the basis of the Lorentz TEM images obtained under thermal fluctuation. I think that it is necessary to clearly show the DPC image of the single elongated skyrmions without merging and the adjacent skyrmions completely divided into two.

Third, as related to the above mentions, the antiskyrmion-mediated creation and annihilation of skyrmions as illustrated in Figs. 4d, 4e cannot be interpreted by the experimental figures only. Basically, these behaviours could not be directly observed by the TEM experiment and are supported by the micromagnetic simulation described in Supplementary Materials, as with Ref. 18. At least, I consider that it is better to put the micromagnetic simulation results in the manuscript.

The other comments:

In Figs. 4d, 4e, the spin textures surrounded by the red square are like antimerons rather than antiskyrmions.

The discussion of 5–7 defects on page 5, lines 12-18 in the manuscript and on page 1, lines 21-28 in Supplementary Materials seem to be incorrect. The authors describe that the total energy is 6% higher and unstable for a 5–7 defect because the energy of a five-fold coordinated skyrmion (5-Sk) is 24% higher than that of a six-fold coordinated skyrmion (6-Sk) and the energy of a seven-fold coordinated skyrmion (7-Sk) is 18% lower than that of the 6-Sk. However, in real FeGe thin films, the triangular skyrmion lattice is the most stable, i.e., the energy of the 6-Sk is the most minimum in the system. Therefore, when the skyrmion arrangement is deviated from the triangular lattice, the energy of such individual skyrmions should be always higher. Basically, the energy of the 5- and 7-Sks is always higher than that of the 6-Sk. Of course, the simulation should output the same results. The reason why the author's micromagnetic simulation output the incorrect results is that the appropriate lattice constant is not set for the triangular skyrmion lattice. It has been reported that, in the micromagnetic simulation, the lattice constant of the triangular skyrmion lattice becomes larger than that of the triple-Q model: (4*pi*A/D)*2/sqrt(3) due to the influence of the magnetic and demagnetizing fields [K. Shibata et al., Phys. Rev. Lett. 118, 087202 (2017)]. In the author's simulation, since the lattice constant of the triangular skyrmion lattice in the domain is smaller than the appropriate lattice constant, the integral energy value of the 7-Sk, of which the size is larger than that of the 6-Sk, is incorrectly lower than that of the 6-Sk.
In addition, when comparing the energies of individual skyrmions, it should be compared not with the integral energy value per the skyrmion, but with integral energy value divided by the volume

of the skyrmion (energy density). For example, if an elongated skyrmion has the same integral energy value with an unelongated (cylindrical or circular) skyrmion, the area of integration in the former is wider than that in the latter. Therefore, the absolute energy density value of the former is smaller than that of the latter. As the result, the former has higher energy than the latter because the calculated energy is negative.
For the above reasons, the authors need to calculate again, and revise the description about comparing the energies.

Part of the caption of Fig. S2 in Supplementary Materials is displayed by white characters.

Dear Reviewers,

We would like to thank you for the time and effort that has been put into reading our manuscript and providing us with valuable feedback. We appreciate your detailed and insightful comments and believe we have been able to improve the manuscript with your suggestions. The point-by-point responses to your comments below include the location of changes within the manuscript (using page number, P, and lines, L) that have been made considering each suggestion.

Reviewer 1:

- *The simulations are performed at zero temperature where the skyrmion tubes are static. In reality they wiggle around which can be seen in the experiments and motivates using the Arrhenius law.*
    - Thank you for this comment. We agree that the processes observed experimentally are thermally activated, and therefore provide motivation for describing the dynamics using the Néel-Arrhenius law. However, the micromagnetic simulation study was undertaken to investigate two key points. Firstly, we wanted to explore the main energy contributions local to 5-7 defects. We believe that this was not clear enough in the original manuscript and have added a clearer description of this point (P:5 L:21-31). Secondly, we wanted to gain insight into the possible mechanistic pathways of skyrmions being created(destroyed) via splitting(merging). The micromagnetic simulations were utilised to probe the possible mechanistic routes during skyrmion merging/splitting, and not the dynamics. We have added a micromagnetic simulations section into Methods, in which we seek to address your comments and clarify our motivations (P:8 L:7-13). Furthermore, the addition of temperature like effects are possible in micromagnetic simulations but it is not realistic to expect that these should provide a digital version of the experiments performed. There is much debate among theoretical colleagues regarding the validity of finite temperature additions in micromagnetics and so we restrict the simulations to areas where the results may be trusted.

- *I am very doubtful if an essentially 2D simulation of the skyrmion lattice is sufficient to capture the underlying physics here. Important, I guess, is the transition of the skyrmion tubes to new positions and this can initialize at various positions within the tube.*
    - Whilst we agree that the ultra-thin films in the simulations cannot give an exact physical description, we believe that they give a strong indication of the pathways undertaken. Our justification for simulating ultra-thin films stems from the fact that our experimental sample thickness was on the order of the helical length of FeGe (70nm) and thus magnetisation variation through the thickness will be minimal (other than the possible existence of the near surface twisting states which help to stabilise the skyrmions over the helical state [Leonov et al., Phys. Rev. Lett. 117, 087202 (2016)]. We have clarified this point on P:6 L:16-18. Additionally, since we have avoided the inclusion of temperature terms in these simulations the thickness must be ultra-thin (see ref 20 in the manuscript). We have clarified this point further in the Methods section (P:8 L:13-14). However, we have added panels to Supplementary Fig. S3f which demonstrates how the antiskyrmion may be destroyed through a larger thickness of 10 nm. Finally, any suggestions provided by micromagnetic simulations of mechanisms occurring through

the thickness are not currently possible for us to confirm, due to the averaged-through-thickness information provided by electron microscopy.

- *Without the comparison to micromagnetics an interpretation soley based on the LTEM images is hardly possible. It is for this reason that I would like to ask the authors to consider more realistic simulations before resubmission to Nature Communications.*
    - We thank the reviewer for their advice. We note that the micromagnetic simulations included in the manuscript assist in interpretation of our Lorentz Fresnel and Lorentz DPC images as well as providing support for our proposed mechanism. As such we have moved the main results (splitting/merging transition states) into the main manuscript in Fig. 4c,d (P:13).

Reviewer 2:

- *I feel Fig. 3b misleading because the coloured broken lines are shown at the tops of the potential and not at the bottoms of the potential which is corresponding to the states.*
    - We agree that the layout of Fig. 3b was potentially misleading and as such we have reworked this figure. The energies corresponding to each energy barrier are now clearly labelled and only given as relative values compared to the most stable observed state, P_1 (see later comment for details on our choice to present only the relative energies). Additionally, the barriers are now scaled to the relative heights in the figure, to demonstrate the relatively small differences in energy for each observed state. See P:12 for the reworked figure.
- *The authors show asymmetric potential barriers in Fig. 3b. According to it, for example, the energy barrier from P1 to T1 is lower than that from T1 to P1. This should result in lower transition probability for transition from P1 to T1 than that for transition from T1 to P2. However, the authors states at p.4, l.32 "Figure 3c also demonstrates the symmetry of the transitions, where transitions to or from a particular state had equal probabilities", which seems to contradict with the transition probabilities expected from the potential barriers. In my opinion, the symmetry of Fig. 3c is just due to appearance and due to the normalization. The authors extracted transition events so that total probability becomes 1. In the present figure, each row is multiplied by the frequency of each state. I suggest normalizing the probability for each row. In addition to it, I think the transition events can be modeled using Markov chain, and I also suggest providing the probability of self-transition, i.e. transition from a state to the same sate, as well.*
    - We would like to thank you for this comment and insightful suggestion. We have now normalised the transition matrix across each row and moved this figure to Supplementary Fig. S1. Additionally, we have added a replacement panel to the reworked Fig. 3 (P:12) which seeks to demonstrate visually, the observed transition lifetimes and probabilities through a Markov Chain diagram. Description of this figure is found on P:5 L:4-7. Unfortunately, it was not possible to include data for self-transitions into this diagram. Temporal resolution in our LTEM Fresnel experiments was limited to 10 ms due to factors involving the available electron flux and the levels of contrast of the skyrmion objects. Our detector permits direct filming at much higher rates, 1200 fps, however in images recorded on these timescales (840 μs) image contrast was compromised by statistical (Poissonic) noise due to the much-reduced intensity levels. This limited our ability to access states

between P_x-T_x or T_x-P_x.

- *In Fig. 4a and 4b, the authors show images of a stable 5-7 defect with explanation of "a static 5-7 defect from a different SkX". In Fig. 4c shows "A Fresnel image of a stable5-7 defect (from the same SkX domain boundary as the dynamic 5-7 defects)". However, the temperature and magnetic field is not shown explicitly for these images. Is the temperature and the magnetic field is the same as the other figures (253 K, 510 Oe)? If so, what is the difference of stable 5-7 defect and dynamic one?*
    - Thank you for this comment. Due to the original layout of Fig. 4, we believe that it was unclear where the Fresnel image in Fig. 4c was obtained from. It was from the same dataset as the Fresnel images in Fig. 2 (further down the SkX boundary). As such we have moved the image of this stable 5-7 defect to Fig. 2 (P:11) where we believe it is more logical to discuss (alongside the dynamic 5-7 defects). Therefore, we have moved the description of this Fresnel image to earlier in the manuscript (P:4 L:9-23). Our explanation for the difference between stable and dynamic 5-7 defects due to experimental and micromagnetic simulations can be found on P:4 L:13-17 and P:5 L:22-32, respectively.

- *In Fig. 4d and 4e, the authors show that the split and merge of skyrmions can be explained in terms of creation and annihilation of antiskyrmion localized at the middle of cores of the two skyrmions. This implies the energy barrier required for the transition takes place by two steps: first step is the deformation of skyrmions (the first red arrow) and the second step is the annihilation of antiskyrmion structure (the second red arrow). The first step seems to be clearly reasonable and to be achieved only by continuous deformation. The second step, however, the transition from the middle state of Fig. 4d and 4e to the last state is not discussed in the manuscript although it is not clear. Concerning this, I found that the only the difference between the middle state of Fig. 4d and 4e is direction of the magnetic moment of the antiskyrmion core. After that, I came to think the transition path from Px to Tx might be like this: Fig.4d initial state -(continuous deformation)-> Fig.4d middle state - (antiskyrmion core flip)-> Fig.4e middle state -(continuous deformation)-> Fig. 4e initial state. And, the transition path from Tx to Px is the opposite. However, I am confused because the flip of the magnetic moment at aitskyrmion core changes the topological chrage by 2, which cannot meet the consistency of the topological charge. Do the authors have any comment of my consideration or any clear answer about the second step?*
    - Thank you for highlighting the need for a fuller explanation on the proposed mechanism. We have added data from the micromagnetic simulations to the manuscript in a reworked Fig. 4 (P:13) to help illustrate the proposed pathways and help with the explanation to and from the middle states in Fig. 4a,b. We have added a more thorough explanation of each step in the proposed processes (P:5 L:42-50 and P:6 L:1-12). We summarise for your information as follows:
        - Splitting (Fig. 4a - P:13): Continuous deformation towards the middle state shown, resulting in two skyrmion structures joined by a central antiskyrmion with the same core polarity as the skyrmions (since it was formed from the central spin-down region of the elongated skyrmion). This middle configuration has the same total topological charge of N = -1 due to the pair creation of skyrmion and antiskyrmion. The second step involves gradual shrinking of the antiskyrmion size (shown in Supplementary Fig. S3e) until it

cannot reduce in size further and the central spin rotates first in-plane (destroying the antiskyrmion topology) and then out of plane, resulting in the two remaining skyrmions with a total N = -2. This is shown by the step change in topological charge tracked in Supplementary Fig. S3b (right panel).

- Merging (Fig. 4b - P:13): Continuous deformation as the inter-skyrmion distance is reduced. This results in a merged elongated skyrmion with a connecting antiskyrmion with opposite core polarity as the skyrmions (since it was formed from the spin-up region of the magnetic moments between the skyrmions, aligned with the external magnetic field). As before, the second step involves gradual shrinking of the antiskyrmion (Supplementary Fig. S3d) until again the spin rotates in-plane destroying the antiskyrmion topology. The result is a single elongated skyrmion with N=-1 and the topological charge change can be seen in Supplementary Fig. S3b (left panel).

- *I understand that even single temperature is enough for just comparing relative energy barrier scales if the observed events obeys the Neel-Arrhenius equation. However, this paper does not provide clear information that the observed events really obeys the Neel-Arrhenius equation. I think it would be better to include data at other several temperatures to support this if possible. Do the authors have any data to support or any comments about this suggestion?*
    - Thank you for this suggestion. It is our assumption that these thermally driven redistributions of magnetisation obey the Neel-Arrhenius law as given in the manuscript to a first approximation. We have added a sentence to clarify this assumption in the manuscript (P:4 L:47-58). Unfortunately, we were not able to investigate the effect of varying the temperature on the observed dynamics. This is because the sample in which these spontaneous events were observed had a narrow phase space region of SkX lattice stability. Therefore a study as a function of temperature would cause regions of the SkX to approach phase transitions altering the energy landscape considerably.

- *The present analysis was done on can the present finding be generally applicable for the general grain boundaries of SkXs, for example, ones with a different misorientation angle?*
    - We have found that generally, an increasing misorientation angle (within the approximate range 0-30 degrees) increases the density of 5-7 defects along grain boundaries, in agreement with other hexagonal lattice systems. We have also identified dynamic (and very often non-repeatable) events occurring at higher rates at these large angle boundaries, the details of which lie outside of practical limits of temporal resolution for these experiments. We have added a sentence to the manuscript in the summary and outlook, addressing this point (P:7 L:6-8).

Reviewer 3:

- *First, it is difficult to understand for me the intention of Figs. 4a-c (the DPC and Lorentz TEM images) to support the author's case as the main result where the creation and annihilation of skyrmions are mediated by antiskyrmions. The reason is that there is no clear description of the observed stationary states such as Px, Tx, intermediate state between Px and Tx. In addition, there is no description how the stationary images were experimentally obtained. Lower temperature than 253 K in Figs. 1, 2? Otherwise, if the stationary images were obtained at 253 K, the high-resolution DPC image makes no sense because of magnetically varying in time during scanning of electron beam.*
    - Thank you for this comment. We believe that the original Fig. 4 was misleading in the way that it was structured. The DPC images show a 5-7 defect (stationary under observation) from a different skyrmion lattice to the LTEM images. The purpose of including the DPC images is to demonstrate the more extreme forms of deformation that can be present and which are made visible when studied with a technique allowing higher spatial resolution (3 nm in this case). We believe that the observed deformations are informative as similar types of deformation are observed, and involved, in the dynamic transformations obtained from micromagnetic simulations. Notably, the deformation and kinking of the SkCoH and regions of in-plane magnetisation surrounding out-of-plane regions. To help to clarify this, we have moved Fig. 4c panel to Fig. 2e (P: 11) and the description of this image to P:4 L: 9-23.

- *Second, this study lacks the confirmed experimental evidence of the existence of both the elongated skyrmions (left in Fig. 4d and right in Fig. 4e) and the adjacent skyrmions completely divided into two (right in Fig. 4d and left in Fig. 4e) as the energy minimum states under thermal fluctuation. The reasons are as follows. As shown in attached Figs. 4a-c that add my comments, in the DPC images (Figs. 4a, b) the SkCoH is no longer single elongated skyrmion because of merging with the adjacent SkCoP and skyrmion. (As mentioned above, it is difficult to understand for me the intention of Figs. 4a-c.) On the other hand, all Lorentz TEM images cannot resolve the above merged state, as exemplified in Fig. 4c. Despite this Lorentz TEM resolution limit, the authors presuppose the existence of both the elongated skyrmions and the adjacent skyrmions, on the basis of the Lorentz TEM images obtained under thermal fluctuation. I think that it is necessary to clearly show the DPC image of the single elongated skyrmions without merging and the adjacent skyrmions completely divided into two.*
    - Thank you for this comment. Further to the comment above, we believe that the original layout of Fig. 4 may have been unclear by linking the Fresnel images to the DPC images. The DPC image shown is not of the same time-varying 5-7 defect shown in the Fresnel images. Therefore, a direct comparison of the details of the magnetisation (as in your attached annotated image) is unfortunately not possible. We have clarified this point further in the written sections (P:6 L:31-32, P:6 L:40-41 and P:7 L:27).

- *Third, as related to the above mentions, the antiskyrmion-mediated creation and annihilation of skyrmions as illustrated in Figs. 4d, 4e cannot be interpreted by the experimental figures only. Basically, these behaviours could not be directly observed by the TEM experiment and are supported by the micromagnetic simulation described in Supplementary Materials, as with Ref. 18. At least, I consider that it is better to put the micromagnetic simulation results in the manuscript.*

- We thank you for your advice to include micromagnetic results in the main manuscript. Alongside comments from all reviewers, we recognise that these should be given prominence due to their importance in our analysis of the proposed mechanism. As such, we have added panels to Fig. 4c, d (P:13) which demonstrate the middle states in Fig. 4a,b as found in our micromagnetic study. As such, we have added a more detailed description of the proposed mechanisms, using these new added figure panels (P:5 L:42-50 and P:6 L:1-12). Finally, we have added a micromagnetics simulation section to Methods, highlighting the main points and assumptions in our simulations (P:8 L:3-16).

- *In Figs. 4d, 4e, the spin textures surrounded by the red square are like antimerons rather than antiskyrmions.*
  - This is partially due to the field of view of the red boxes in Fig. 4a,b (P:13). There is out-of-plane regions directly above and below the boxed regions shown in the middle images of Fig. 4a,b. The identification of these regions as antiskyrmion is futher exemplified by the integer change in topological charge when these objects are destroyed (Supplementary Fig. S3b).

- *The discussion of 5–7 defects on page 5, lines 12-18 in the manuscript and on page 1, lines 21-28 in Supplementary Materials seem to be incorrect. The authors describe that the total energy is 6% higher and unstable for a 5–7 defect because the energy of a five-fold coordinated skyrmion (5-Sk) is 24% higher than that of a six-fold coordinated skyrmion (6-Sk) and the energy of a seven-fold coordinated skyrmion (7-Sk) is 18% lower than that of the 6-Sk. However, in real FeGe thin films, the triangular skyrmion lattice is the most stable, i.e., the energy of the 6-Sk is the most minimum in the system. Therefore, when the skyrmion arrangement is deviated from the triangular lattice, the energy of such individual skyrmions should be always higher. Basically, the energy of the 5- and 7-Sks is always higher than that of the 6-Sk. Of course, the simulation should output the same results. The reason why the author's micromagnetic simulation output the incorrect results is that the appropriate lattice constant is not set for the triangular skyrmion lattice. It has been reported that, in the micromagnetic simulation, the lattice constant of the triangular skyrmion lattice becomes larger than that of the triple-Q model: (4*pi*A/D)*2/sqrt(3) due to the influence of the magnetic and demagnetizing fields [K. Shibata et al., Phys. Rev. Lett. 118, 087202 (2017)]. In the author's simulation, since the lattice constant of the triangular skyrmion lattice in the domain is smaller than the appropriate lattice constant, the integral energy value of the 7-Sk, of which the size is larger than that of the 6-Sk, is incorrectly lower than that of the 6-Sk.*
  In addition, when comparing the energies of individual skyrmions, it should be compared not with the integral energy value per the skyrmion, but with integral energy value divided by the volume of the skyrmion (energy density). For example, if an elongated skyrmion has the same integral energy value with an unelongated (cylindrical or circular) skyrmion, the area of integration in the former is wider than that in the latter. Therefore, the absolute energy density value of the former is smaller than that of the latter. As the result, the former has higher energy than the latter because the calculated energy is negative.
  For the above reasons, the authors need to calculate again, and revise the description about comparing the energies.
  - We would like to thank you for this detailed suggestion and insight. We have since modified the simulations with appropriate skyrmion lattice parameter and all

energies calculated are energy densities. See Supplementary Note 2 for these changes and a reworked figure, Fig. S2.

- *Part of the caption of Fig. S2 in Supplementary Materials is displayed by white characters.*
    - This has been fixed in the rework of Fig. S2.

Reviewers' Comments:

Reviewer #1:
Remarks to the Author:
The authors went through great efforts and in my opinion have improved the manuscript. I still think that the findings are a bit speculative since they are based on essentially a two dimensional treatment at zero temperature of a three dimensional problem at elevated temperatures (even though the authors mention in the manuscript and in the rebuttal that they do not expect the magnetization profile to change much across the film thickness). This makes me less convinced that the authors indeed found a new mechanism (antiskyrmion generation and annihilation vs. “zippers”), but it may well be possible. I just think that the paper does not provide a stringent proof.
But, as I have mentioned in my first report, the data is of high quality and the discussion is adequate. So probably this paper brings something new for the experts in the field. I still think that the mechanism is so similar to the one observed for vortex core reversal where the same problem has to be discussed, namely the conservation of topological charge, that I think this work should be cited. Quite a few papers also discuss the energetic contributions to this very similar reversal process and in fact figure 4 resembles the sketch known for vortex core reversal.

In my opinion this a bit a borderline case since in my view the (reasonable) arguments that the authors bring forward are not 100% backed by the experiments for the reasons mentioned above. I leave it to the editor to decide.

Reviewer #2:
Remarks to the Author:
I am satisfied that the authors have addressed my comments carefully. The revised explanation and the modified discussion sufficiently provide valuable data set, information for reproducibility, and interesting insight. The interpretation of data and suggested transition mechanism are based on some assumptions, but they are plausible at the present point. Thus, I now recommend the manuscript for publication.

Reviewer #3:
Remarks to the Author:
The resubmitted manuscript addresses my questions and doubts correctly enough, though I don't feel like it's worthy enough for the DPC data to put it in the main text. So, I can recommend publication in Nature Communications.

Dear Reviewers,

We would like to thank you again for the time and effort that has been put into reading and assessing our revised manuscript. The point-by-point responses to any final comments is given below, including the location of changes within the manuscript (using page number, P, and lines, L) that have been made considering each suggestion.

Reviewer 1:

- *The authors went through great efforts and in my opinion have improved the manuscript. I still think that the findings are a bit speculative since they are based on essentially a two dimensional treatment at zero temperature of a three dimensional problem at elevated temperatures (even though the authors mention in the manuscript and in the rebuttal that they do not expect the magnetization profile to change much across the film thickness). This makes me less convinced that the authors indeed found a new mechanism (antiskyrmion generation and annihilation vs. "zippers"), but it may well be possible. I just think that the paper does not provide a stringent proof.*
  But, as I have mentioned in my first report, the data is of high quality and the discussion is adequate. So probably this paper brings something new for the experts in the field. I still think that the mechanism is so similar to the one observed for vortex core reversal where the same problem has to be discussed, namely the conservation of topological charge, that I think this work should be cited. Quite a few papers also discuss the energetic contributions to this very similar reversal process and in fact figure 4 resembles the sketch known for vortex core reversal.

  In my opinion this a bit a borderline case since in my view the (reasonable) arguments that the authors bring forward are not 100% backed by the experiments for the reasons mentioned above. I leave it to the editor to decide.
    - Thank you for bringing the vortex core reversal mechanism to our attention. We agree that there is enough similarity that the work in this area should be cited in our discussion of the creation/annihilation mechanisms. We have therefore added a sentence and reference to the manuscript (P:6 L:24-26).

Reviewer 2:

- *I am satisfied that the authors have addressed my comments carefully. The revised explanation and the modified discussion sufficiently provide valuable data set, information for reproducibility, and interesting insight. The interpretation of data and suggested transition mechanism are based on some assumptions, but they are plausible at the present point. Thus, I now recommend the manuscript for publication.*
    - We would like to thank you for your recommendation to publish our manuscript.

Reviewer 3:

- *The resubmitted manuscript addresses my questions and doubts correctly enough, though I don't feel like it's worthy enough for the DPC data to put it in the main text. So, I can recommend publication in Nature Communications.*

- Thank you for your recommendation to publish our manuscript and your suggestions regarding the DPC data. However, we believe that the DPC panels in Fig. 4 are valuable for demonstrating the level of deformation of 5-7 defects with a high spatial resolution. The DPC images indicate regions of connected in-plane magnetisation and isolated out-of-plane magnetisation between skyrmions. According to the micromagnetic simulations both of these properties are important for the creation/annihilation mechanisms involving antiskyrmions. We have decided therefore, to leave the DPC panels in the manuscript.
